# A Case of Amyopathic Dermatomyositis with Pneumomediastinum and Subcutaneous Emphysema

**DOI:** 10.1155/2015/813902

**Published:** 2015-10-18

**Authors:** Aslıhan Gürün Kaya, Aydın Çiledağ, Orhan Küçükşahin, Özlem Özdemir Kumbasar, Çetin Atasoy

**Affiliations:** ^1^Department of Chest Diseases, Ankara University School of Medicine, Ankara, Turkey; ^2^Department of Rheumatology, Ankara University School of Medicine, Ankara, Turkey; ^3^Department of Radiology, Ankara University School of Medicine, Ankara, Turkey

## Abstract

A 34-year-old man was admitted with dyspnea, cough, and fever. Thorax computed tomography revealed ground glass opacities and pneumomediastinum. The patient was diagnosed as amyopathic dermatomyositis due to skin lesions and radiological findings. Despite immunosuppressive treatment clinical deterioration and radiological progression were observed and the patient died because of severe hypoxemic respiratory failure. The patient presented with extremely rare occurrence of pneumomediastinum and subcutaneous emphysema in amyopathic dermatomyositis with a poor prognosis.

## 1. Introduction

Dermatomyositis (DM) is an inflammatory disease characterized by skin lesions with muscle weakness. Amyopathic dermatomyositis (ADM) is a subtype of DM with characteristic cutaneous manifestations but without muscle involvement. Respiratory diseases, especially interstitial lung diseases (ILD), present more than half of the patients. Several reports have documented cases of interstitial lung diseases and the most common histological findings are nonspecific interstitial pneumonia, organising pneumonia, diffuse alveolar damage, and usual interstitial pneumonia. Pneumomediastinum, pneumothorax, and subcutaneous emphysema are rare, but characteristic complications with poor prognosis [1, 2].

## 2. Case Report

**Figure 1:**
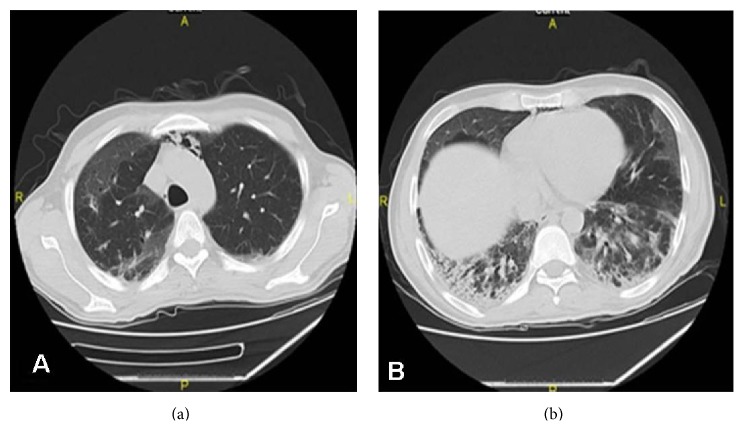
Thorax CT revealing bilateral ground glass opacity in subpleural areas, consolidation, and pneumomediastinum.

A 34-year-old male patient was admitted with dyspnea, cough, and fever for two weeks. There was no history of any occupational exposure and he had a 10-pack-year smoking history. The patient had a diagnosis of undifferentiated arthritis four months ago. He received 21 days of salazopyrin treatment but the drug had been discontinued because of hepatotoxicity. The symptoms were developed after 3 months of the cessation of the drug. On physical examination, cyanosis, macular erythema at neck and extensor surfaces of the arms, and also Gottron papules in joints and dorsum of the hand were detected and bilateral fine crackles were heard. The erythrocyte sedimentation rate was 70 mm/h and CRP level was 32.3 mg/dL. Serum biochemistry and complete blood count were normal. ANA, ANCA, RF, and other immune markers were negative, while anti-SS-A(Ro) was positive. The smear and culture of sputum were negative for any microorganism and acid-fast bacilli. Chest X-ray revealed bilateral opacities and computed tomography (CT) showed bilateral ground glass opacity in subpleural areas, consolidation, and pneumomediastinum (Figure 1).

The patient was transferred to intensive care unit (ICU) due to severe hypoxemic respiratory failure. There was no clinical and radiological improvement despite broad-spectrum antibiotics. Corticosteroid treatment was started because of severe hypoxemic respiratory failure. He did not complain of dry eye and/or mouth. Schirmer's test was negative and biopsy of salivary gland revealed minimal chronic inflammation. Serum creatine kinase (CK) and aldolase levels were normal. The patient was accepted as ADM due to skin lesions without myositis and radiological findings.

**Figure 2:**
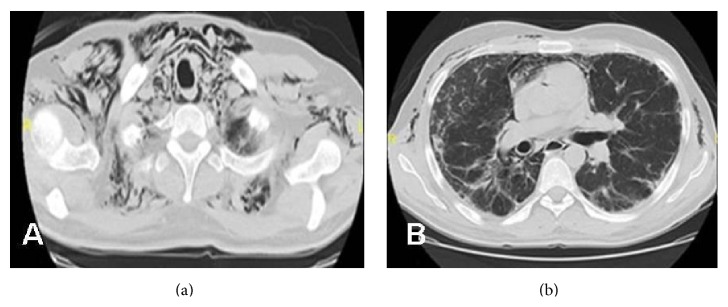
Thorax CT revealing pneumomediastinum, interstitial and subcutaneous emphysema, thickening of interlobular septa, and reticular pattern.

**Figure 3:**
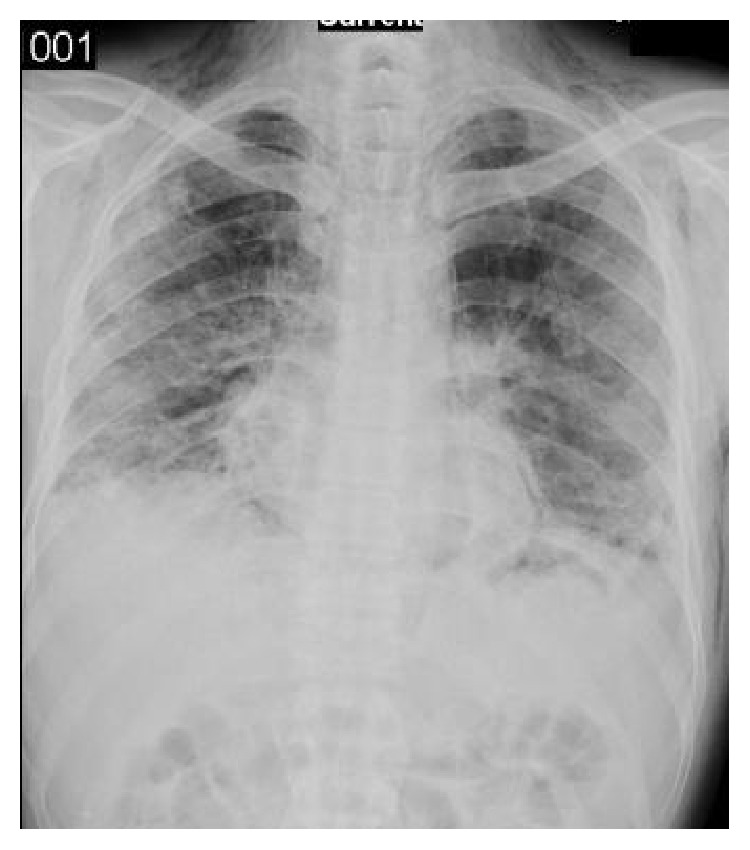
Chest radiograph showing bilateral opacities, pneumomediastinum, and subcutaneous emphysema.

Cyclophosphamide 600 mg/m^2^per 21 days, methylprednisolone 60 mg/day, and hydroxychloroquine 400 mg/day were initiated. A biopsy of skin lesion was performed. Although pathologic examination revealed no specific pathology, we suggested that this might be secondary to the corticosteroid treatment. After second course of cyclophosphamide, although a clinical improvement was observed, chest radiograph and CT showed a progression with pneumomediastinum, interstitial and subcutaneous emphysema, thickening of interlobular septa, and reticular pattern (Figures 2 and 3). Cyclophosphamide treatment was discontinued, the dose of corticosteroids was increased, and mycophenolate mofetil (MMF) treatment was started. The patient was discharged with MMF and corticosteroid therapy. One month later, he was admitted with severe dyspnea on second admission. A radiological progression was observed. He was hospitalized in the ICU; however, the patient died because of severe hypoxemic respiratory failure despite immunosuppressive treatment and mechanical ventilation.

## 3. Discussion

Dermatomyositis is an inflammatory disease with characteristic cutaneous and musculoskeletal findings. Amyopathic dermatomyositis is a clinical subtype of DM and represents an estimated 20% of all DM cases [3]. It was first described by Pearson in 1979 as a rare skin disease that has the same cutaneous symptoms as classic DM without myopathy [4].

Onset usually occurs in early adulthood, although juvenile forms have been reported. The disease is more common in female. In a report including 291 adult-onset ADM cases, 73% of patients were female [5].

The diagnostic criteria for diagnosis of ADM vary. In 1993, Euwer and Sontheimer published four diagnostic criteria for ADM; (1) cutaneous changes pathognomonic of DM, (2) skin biopsy specimen findings compatible with DM, (3) absence of clinical evidence of proximal motor weakness within 2 years of skin disease, and (4) normal skeletal enzyme levels for 2 years following the appearance of skin lesions [6]. However, Stonecipher et al. described different criteria and they divided patients with ADM into 3 main groups: (1) patients with cutaneous changes only, (2) patients with cutaneous changes at baseline and subsequent evolution to myositis, and (3) patients with cutaneous changes and normal muscle enzyme levels but in whom diagnostic evaluations showed subclinical myositis [7]. Also, Sontheimer et al. reported several major and minor criteria for the diagnosis of ADM and in our case the diagnosis was made according to presence of these two minor criteria and one major criterion [8]. Although the skin biopsy revealed no specific pathology in presented case, the biopsy had been performed under corticosteroid treatment.

The cutaneous findings in ADM are not different from those in classic D. Heliotrope rash and Gottron's papules are the most common skin changes. El-Azhary and Pakzad retrospectively reviewed 37 ADM cases and reported that Gottron's papules on the dorsa of the hands appear to be the most consistent presentation [9].

While the patients with DM-associated ILD are more likely to have the antiaminoacyl transfer RNA synthetase antibody (anti-Jo-1), this antibody is typically absent in patients with ADM even when ILD is present as in our case. In the study performed by El-Azhary and Pakzad, antinuclear antibodies (ANA) were positive in 20 of 28 patients [9]. In another report, ANA was positive in 2 of 8 ADM cases [10]. In 2005, Sato et al. reported a newly specific autoantibody in ADM patients which was named as anti-CADM-140 antibody [11]. This antibody may be detected in 50–73% of ADM patients. Gerami et al. reported that, in 72 of 115 ADM patients, ANA was positive (63%), while anti-Jo-1 antibodies were positive in 3 of 85 cases (4%) [5]. They also reported that assays for specific ANAs such as double-stranded DNA, Sm, Ro, and La were negative when reported. In contrast, in our case, only anti-SS-A(Ro) was positive, and, to our knowledge, this is the first case which is compatible with ADM with a positive anti-SS-A(Ro). We could not analyze the anti-CADM-140 antibody because of absence of this test in our laboratory.

Pulmonary involvement is seen in up to 50% of ADM patients and the most common radiographic abnormality is a diffuse reticulonodular pattern with patchy bilateral ground glass opacities. Spontaneous pneumomediastinum and subcutaneous emphysema are very rare and only several cases have been reported previously. Pulmonary involvement is the main cause of death in ADM and severe ILD and pneumomediastinum are the most potentially life-threatening complications. The presence of spontaneous pneumomediastinum is important, because it often leads to a rapid and aggressive course with fatal prognosis [1]. The rupture of subpleural blebs and cysts that developed from interstitial fibrosis and a raised intra-alveolar pressure have been speculated as potential risk factors for development of pneumomediastinum. It has also been reported that corticosteroid treatment may lead to pneumomediastinum by weakening alveolar walls [12, 13].

There is no standard treatment strategy for ADM pulmonary complication. Cozzani et al. reviewed 55 ADM cases with pulmonary involvement and reported that high dose systemic corticosteroids were the first line therapy in all cases. Immunosuppressive or immunomodulant agents, like azathioprine, methotrexate, cyclosporine A, cycles of cyclophosphamide, high dose intravenous immunoglobulin, tacrolimus, or mycophenolate mofetil, were associated in 46 patients as first line therapy and in five cases as second line therapy [1]. The authors also reported a case of ADM with lung involvement in whom a successful outcome was achieved by mycophenolate mofetil without concomitant high dose corticosteroids. The efficacy of mycophenolate mofetil in ILD complicating ADM has also been shown in a few reports [14, 15]. Also, the potential effectiveness of an intensive therapy protocol with the addition of calcineurin inhibitor to glucocorticoid or more drug combination has been reported. (x) MMF suppresses lymphocyte proliferation via the inhibition of inosine monophosphate dehydrogenase, a rate-limiting enzyme for the de novo synthesis of guanosine nucleotides, and inhibits fibrosis via direct suppression of fibroblast function. In our case, initially we started with methylprednisolone, hydroxychloroquine, and cyclophosphamide. Due to absence of an improvement, the therapy was switched to corticosteroid and mycophenolate mofetil. Unfortunately, no improvement was observed and, after a rapid progression, the patient died.

In conclusion, since a rapidly progressive and fatal ILD with pneumomediastinum may develop, ADM cases must be followed closely. An effective and optimal treatment strategy is still needed.
